# Comprehensive evaluation of vaginal intraepithelial neoplasia development after hysterectomy: insights into diagnosis and treatment strategies

**DOI:** 10.1007/s00404-024-07530-1

**Published:** 2024-05-14

**Authors:** Jiahui Wei, Yumei Wu

**Affiliations:** Beijing Obstetrics and Gynecology Hospital, Capital Medical University. Beijing Maternal and Child Health Care Hospital, Beijing, China

**Keywords:** Vaginal intraepithelial neoplasia, Hysterectomy, Clinical features, Diagnosis, Management

## Abstract

Vaginal intraepithelial neoplasia (VaIN), a precancerous lesion associated with human papillomavirus (HPV), impacts women’s health and quality of life. However, the natural progression of VaIN after hysterectomy remains uncertain, due to its low incidence. The existing literature predominantly consists of single-center retrospective studies lacking robust evidence-based medicine. The management of VaIN after hysterectomy is diverse and controversial, lacking a consensus on the optimal approach. Therefore, it is imperative to investigate the development of VaIN after hysterectomy, emphasizing the importance of accurate diagnosis and effective management strategies.

## What does this study add to the clinical work

**Table:** 

The natural history and potential evolution into invasive cancer of vaginal intraepithelial neoplasia after hysterectomy are uncertain. So far, there is still no consensus regarding its diagnosis and management. This review aims to provide a comprehensive overview of existing research on vaginal intraepithelial neoplasia after hysterectomy, with the goal of raising attention and improving management strategies.	

## Introduction

Graham and Meigs first reported the concept of vaginal intraepithelial neoplasia (VaIN) in 1952 [1]. VaIN refers to different degrees of atypical hyperplasia confined to the vaginal epithelium, which is mostly a precancerous lesion of vaginal invasive carcinoma, and often exists concurrently with vulvar and cervical intraepithelial lesions [2]. In the 4th edition of the Classification of Tumors of the Female Genital Organs published by the World Health Organization in 2014, a 2-tiered classification of VaIN was adopted. The new classification system now includes two categories: low-grade squamous intraepithelial lesion (LSIL) and high-grade squamous intraepithelial lesion (HSIL), which replace the previous 3-tiered classification of VaIN I, VaIN II, and VaIN III [3]. As clinicians may have different levels of acceptance towards the revised nomenclature, both vaginal LSIL (VaIN I) and vaginal HSIL (VaIN II/III) will coexist during the transition phase. Initial studies have indicated a low occurrence of VaIN, with an annual incidence ranging from 0.2 per 100,000 to 2 per 100,000. In addition, VaIN was found to account for only 0.4% of pre-cancerous lesions in the lower genital tract of women [4, 5]. With the continuous improvement of cytology, HPV screening, and colposcopy techniques, the annual detection rate of VaIN has been increasing [6]. Schockaert et al. found that the incidence of VaIN II +  was as high as 7.4% in patients who underwent hysterectomy for CIN II +  [7]. VaIN after hysterectomy is often found in the vaginal stump and both parietal horns, and it is usually multifocal and difficult to detect [8]. Despite the increasing interest in the clinical significance of VaIN after hysterectomy, our understanding of its natural progression, effectiveness of treatment, and risk of recurrence or progression remains limited. Therefore, this review aims to provide a comprehensive overview of the clinical features, diagnosis, and treatments of VaIN after hysterectomy.

## Risk factors

While VaIN and CIN share similar risk factors, further research is needed to understand the internal molecular pathogenesis of VaIN after hysterectomy. Several studies have indicated that the risk of vaginal HSIL progressing to cancer varies between 2 and 12% [9].

### Persistent high-risk HPV infection

Bryan et al. found that HPV infection was detected in 96% of patients diagnosed with VaIN [10]. A retrospective analysis conducted at the West China Second Hospital, involving 3229 patients with histopathologically confirmed VaIN, revealed that two-thirds of the patients were infected with HPV16, and the severity of VaIN grading showed a positive correlation with the rate of HPV16 positivity [11]. Bogani et al. confirmed that post-treatment HPV persistent infection and pre-treatment HPV-31 infection were identified as risk factors in the recurrence of vaginal HSIL [12]. In addition, the stability and composition of vaginal microbiota can influence the viral infection status by modulating the immune system in the female lower genital tract [13]. A reduction in lactobacillus species has been associated with HR-HPV infection [14, 15].

### History of cervical cancer and cervical intraepithelial neoplasia

The upper one-third of the vagina and the cervix have the same embryological origin, as they both develop from the genitourinary sinus and exist in a similar physiological environment [16]. In a retrospective analysis by Dan et al., a total of 8581 patients who underwent hysterectomy were examined. The study revealed a significant difference in the incidence of VaIN, with rates of 7.3% and 0.3% observed in patients with and without a history of cervical intraepithelial neoplasia (CIN), respectively [17]. It is important to highlight that approximately 1% to 7% of patients who undergo hysterectomy for CIN or cervical cancer may develop VaIN, typically within a period of 2 years [5, 6]. Therefore, it is recommended to perform routine vaginal wall biopsy under colposcopy prior to surgery for patients undergoing hysterectomy for stage IA cervical cancer or CIN III, which aids in determining the necessary extent of vaginal resection during the procedure. Furthermore, regular follow-up on the vaginal stump after hysterectomy is advised to prevent the progression of VaIN.

### History of radiation therapy

In patients who undergo vaginal radiotherapy after hysterectomy, the incidence of vaginal HSIL is twice as high compared to patients without a history of radiotherapy, and it often occurs 10 to 15 years after radiotherapy [18]. Radiotherapy can cause vaginal epithelial atrophy, congestion, edema, and reduced-mucosal resistance, increasing susceptibility to HPV infection, which is a possible main cause of VaIN. In vitro studies have shown that radiation induces the expression of HPV oncoproteins and MHC class I restriction elements in cervical cancer cells, potentially contributing to the development of vaginal cell dysplasia after radiation [19].

### Other factors

Factors such as immune deficiency, organ transplantation, smoking, age, early sexual intercourse, multiple sexual partners, multiple pregnancies, educational level, and economic status have also been identified to be associated with the occurrence and progression of VaIN after hysterectomy [2, 20].

## Clinical manifestations

**Fig. 1:**
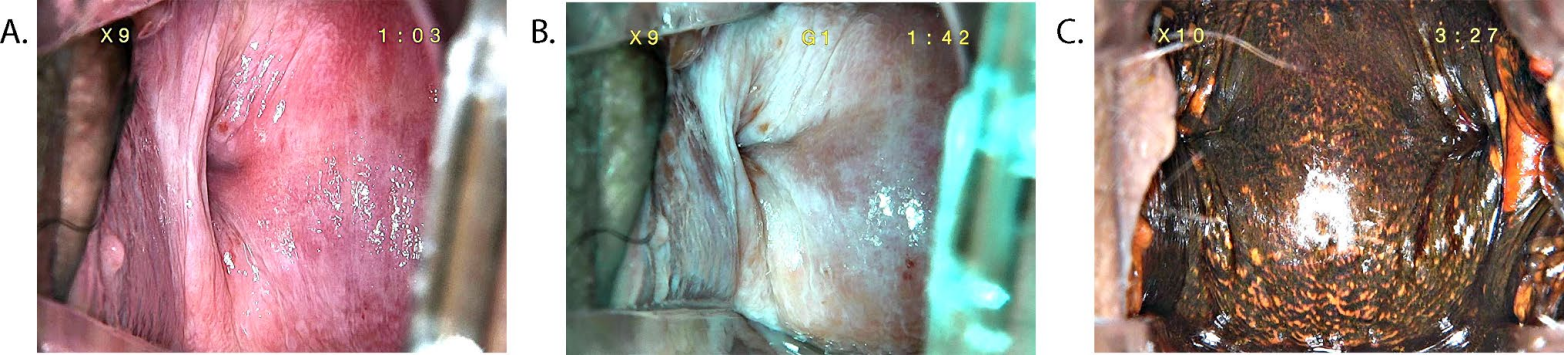
Vaginal LSIL after hysterectomy. (**A**) Macroscopic view under colposcopy. (**B**) View under green filter. (**C**) Iodine staining test view under colposcopy

**Fig. 2:**
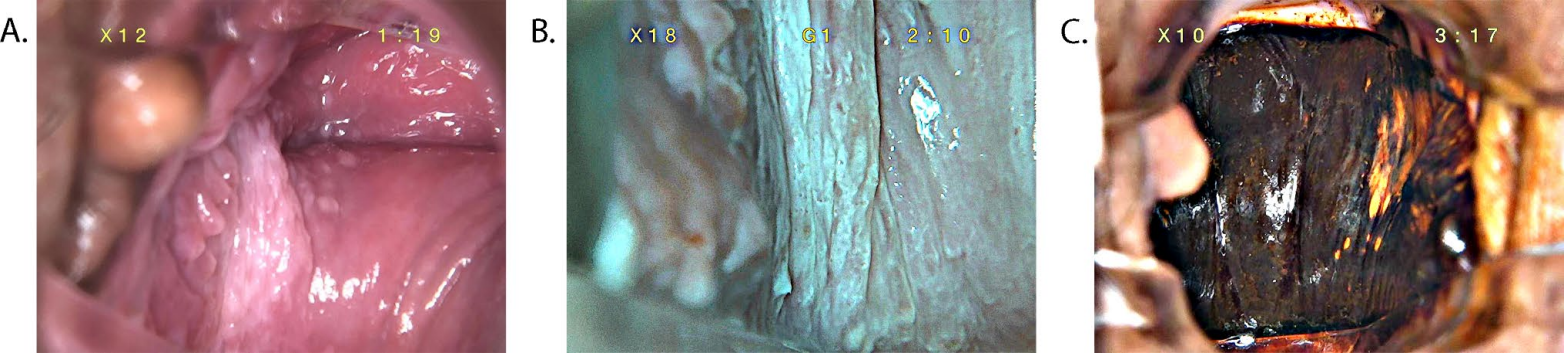
Vaginal HSIL after hysterectomy. (**A**) Macroscopic view under colposcopy. (**B**) View under green filter. (**C**) Iodine staining test view under colposcopy

**Fig. 3:**
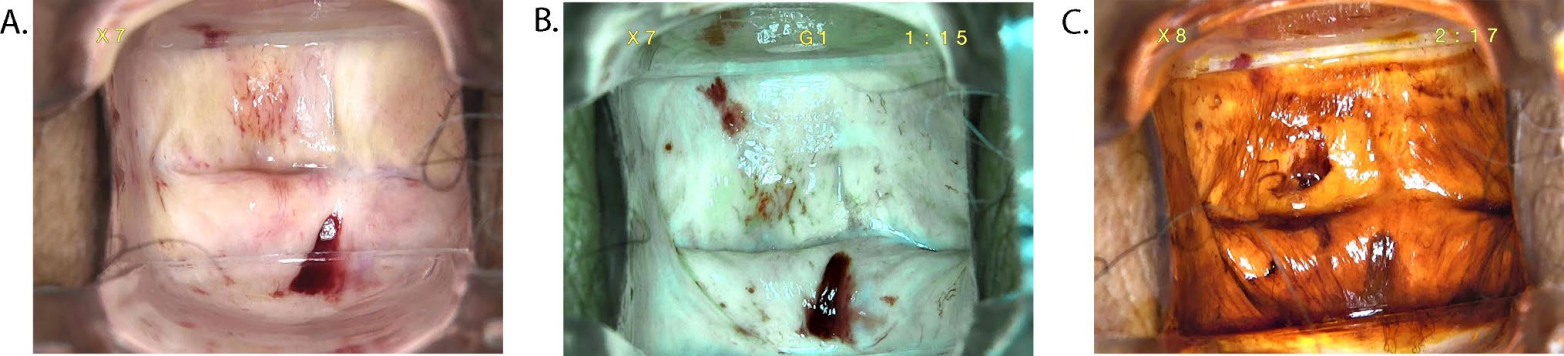
Vaginal LSIL after postoperative radiotherapy and chemotherapy for cervical cancer. (**A**) Macroscopic view under colposcopy. (**B**) View under green filter. (**C**) Iodine staining test view under colposcopy

**Fig. 4:**
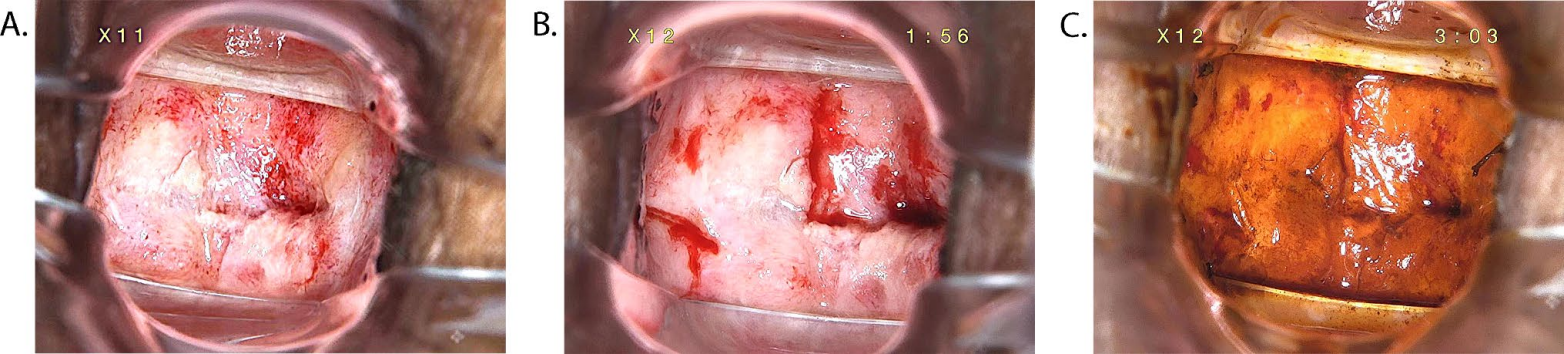
Vaginal HSIL after postoperative radiotherapy and chemotherapy for cervical cancer. (**A**) Macroscopic view under colposcopy(× 11 times). (**B**) Macroscopic view under colposcopy(× 12 times). (**C**) Iodine staining test view under colposcopy

Most VaIN patients have no noticeable symptoms. As the condition worsens, vaginal bleeding and increased secretions can be observed in a small number of patients [5]. During gynecological examinations, the vaginal stump mucosa may appear smooth, slightly eroded, or rough. In colposcopy, it often shows dense and thick acetowhite epithelium, positive iodine tests in certain cases, and thick and uneven punctate blood vessels (Figs. 1, 2, 3 and 4).

## Auxiliary examination and diagnostic methods

### Vaginal cytology screening

Patients with VaIN often seek medical treatment following abnormal cytology screening. He et al. found a significant correlation between cervical lesions and vaginal lesions, suggesting that cytology screening can be used as a routine method for screening both cervical and vaginal diseases. In cases where hysterectomy is performed for cervical cancer or CIN, vaginal cytology screening can help enhance the detection rate of VaIN, and special attention should be given to the location of specimen collection during the operation [21]. Grace et al. conducted a study on the routine use of vaginal vault cytology in surveillance after hysterectomy for early stage cervical cancer. Their findings indicated that this practice does not seem to affect the detection of recurrent malignancy [22]. Stokes-Lampard et al. conducted a retrospective study on 6543 patients who underwent hysterectomy for benign diseases and found that 1.8% of patients had cytological abnormalities, 0.12% of patients had histological abnormalities, and no cases of malignant tumors. It is believed that routine vaginal cytology examination is not necessary after hysterectomy for benign diseases, but will cause anxiety to patients and waste of public resources [23]. According to Frega et al., a study showed that 3.9% of patients who underwent a hysterectomy for benign lesions developed VaIN. The researchers recommended that routine cytology screening should be carried out for patients who have had a hysterectomy for benign lesions. Furthermore, it was observed that among patients with VaIN recurrence, there was a notable increase in the positive rate of high-risk HPV (16 or 18) [24]. In conclusion, the vaginal cytology screening for patients who have undergone hysterectomy for cervical cancer or CIN is advisable. This is important to detect the occurrence of VaIN at an early stage. There is ongoing debate regarding whether patients who undergo hysterectomy for benign diseases should undergo postoperative cytology screening. Considering a few patients may still have persistent HPV infection and the potential for VaIN, it is also recommended that these patients undergo regular vaginal cytology screening to promptly identify VaIN in the stump.

### High-risk HPV testing

Over 100 different types of HPV have been detected, which can be classified as high-risk human papillomavirus (HR-HPV) or low-risk human papillomavirus (LR-HPV) based on their carcinogenicity [25]. In recent years, there has been a noticeable increase in the rate of HPV infections, which has consequently led to a rise in the incidence of VaIN after hysterectomy. Current research indicates that the persistent infection of HR-HPV is the primary cause of VaIN. Approximately 90% of VaIN patients have tested positive for HPV, with 70% being infected with HR-HPV and 30% with LR- HPV. Among the HR-HPV types, HPV16, 43, 51, 52, 53, 56, 58, and 59 are the most commonly found [26, 27]. The positivity rate of HPV is positively correlated with histological grade, and high-risk HPV typing is valuable for diagnosing and predicting the outcome of VaIN patients, particularly HPV16. Among VaIN I-III and vaginal cancer, HPV16 has the highest positive rate [11]. Bogani et al. conducted a retrospective analysis of 77 cases of vaginal HSIL and found that HPV31 infection might be a risk factor for VaIN recurrence [12]. HR-HPV testing has a higher sensitivity in detecting VaIN after hysterectomy than cytology screening, and combined screening using both methods can increase the accuracy to 95% [28]. Ao et al. analyzed 1932 cases of VaIN and found cytology screening combined with HPV testing could increase the screening sensitivity to 98.1% [11]. In conclusion, HR-HPV testing holds significant value in the diagnosis and prediction of VaIN after hysterectomy, while also serving as a means to verify the accuracy of cytology screening. Combining HR-HPV testing with cytology screening can enhance the sensitivity and specificity of VaIN after hysterectomy screening. Furthermore, HR-HPV typing may aid in risk stratification, disease progression prediction, and treatment guidance for VaIN. Nevertheless, there is currently a debate regarding the predictive value of HPV quantitative testing for the outcome of VaIN after hysterectomy.

### Indications and precautions for colposcopy

Approximately two-thirds of VaIN cases occur in the upper one-third of the vagina, often at the sutures of the vaginal stump. Given that VaIN after hysterectomy is typically multifocal, colposcopy plays a crucial role in detecting and monitoring diseases. Abnormal colposcopy images of VaIN after hysterectomy usually exhibit micropapillary hyperplasia, acetowhite epithelium, punctate blood vessels, and iodine-unstained epithelium. The characteristic appearance of these abnormal images becomes more pronounced with higher levels of VaIN. Scattered and punctate lesions often indicate vaginal LSIL, whereas large single lesions typically indicate vaginal HSIL [29]. The reliability of the 2011 International Federation of Cervical Pathology and Colposcopy (IFCPC) colposcopy terminology ranged from 69.2% to 82.5% [2]. After hysterectomy, particularly in patients who have undergone the procedure due to cervical lesions, it is essential to conduct colposcopy when vaginal cytology is abnormal or HR-HPV infection persists to assess the severity of vaginal lesions. To avoid missing the diagnosis of VaIN after hysterectomy, colposcopy should be performed on high-risk patients. The indications for colposcopy include: (1) abnormal vaginal discharge or vaginal bleeding; (2) presence of vaginal stump vegetations or vaginal wall tumors; (3) history of hysterectomy due to cervical cancer or cervical intraepithelial neoplasia; (4) abnormal vaginal stump cytology; (5) persistent HR-HPV infection or infection with HPV type 16 or 18; (6) history of VaIN; (7) history of genital warts; (8) history of radiotherapy for cervical cancer [5, 19, 26, 30–32]. To address vaginal stenosis, atrophy, few folds, and poor elasticity in patients who have undergone hysterectomy combined with radiotherapy or postmenopausal hysterectomy patients, it is recommended to topically apply estrogen ointment for a period of 2–4 weeks [33]. To detect VaIN or early vaginal invasive cancer, we suggest performing transvaginal suturing of the stump, allowing for a thorough examination of the vaginal epithelium through colposcopy. When suturing the vagina laparoscopically and abdominally, it is important not to suture both ends of the vaginal apex to the stump of the cardinal ligament, as this can lead to the formation of vaginal crypts, which cause difficulties in subsequent colposcopy and biopsy.

### P16/Ki-67 double-staining detection

P16 is a protein that regulates the cell cycle and induces cell cycle arrest under normal physiological conditions. Ki-67 is a marker of cell proliferation. Generally, p16 and Ki-67 are not co-expressed in the same cervical epithelial cells. Therefore, detecting co-expression of p16/Ki-67 can be an effective marker for cell cycle dysregulation caused by HR-HPV infection [34]. Previous studies have shown that p16/Ki-67 double staining has a sensitivity of 91.6% and specificity of 95.0% in detecting VAINII +  [35]. Boonlikit et al. found that compared to cytology screening, p16/Ki-67 double staining can be an effective triage strategy for HR-HPV-positive women [36].

## Treatments

The treatment of VaIN after hysterectomy is controversy. Due to the low incidence of VaIN, it is challenging to conduct prospective studies that compare the effects of different treatment methods, resulting in a lack of standardized treatment protocols. In cases of vaginal LSIL with a low risk of recurrence, close observation and follow-up may be sufficient. However, for vaginal HSIL or VaIN associated with CIN or cervical cancer, which have a higher risk of progression to invasive cancer and a higher recurrence rate, active medical intervention is recommended. The choice of VaIN treatment plan should be individualized, taking into consideration factors such as patients’ age, physical condition, medical history, lesion location, and desire to preserve sexual function. Prior to initiating any treatment, it is crucial to conduct a comprehensive colposcopy to ensure that no lesions are missed. In addition, it is essential to fully educate patients about the potential complications or adverse reactions that may arise from each treatment plan. Surgical excision is the primary treatment approach, particularly when infiltration cannot be ruled out. Topical medications are suitable for women who have persistent, multifocal disease or are unable to undergo surgery. Brachytherapy, despite its association with high morbidity, may be considered for women with multifocal disease who are not suitable candidates for surgery or have not responded to other treatments. CO_2_ laser ablation can effectively minimize scarring and sexual dysfunction. It is worth noting that no treatment has been proven to be superior to any other in terms of recurrence rates (Table 1) [37–39].

**Table 1:** Different treatment modalities of VaIN after hysterectomy

Treatment Author, (reference)	Stage	Patients (*n*)	Study type	Effective rate (%)	Advantages	Disadvantages
**Imiquimod**					Easy to operate and does not affect Vaginal function	The efficacy is unclear
Inayama et al. [40]	VaIN II–III	37	Meta-analysis	76%		
Haidopoulos et al. [41]	VaIN II–III	7	Prospective	57%		
Buck et al. [42]	VaIN I–III	42	Prospective	86%		
**Topical 5-FU**						
Fiascone et al. [45]	VaIN II–III	47	Retrospective	74%		
Rome et al. [46]	VaIN I–III	11	Retrospective	46%		
**Trichloroacetic acid, 50%**						
Lin et al. [49]	VaIN I–III	28	Retrospective	53%VaINII-III 100% VaINI		
**CO**_**2**_** laser**					Simple operation, the effect on vaginal function is small, and the treatment can be repeated	No pathological results, the vaginal stump lesions are not easily exposed, and the depth of treatment is limited
He et al. [50]	VaIN II–III	116	Retrospective	75%		
**Photodynamic therapy**					Safe and simple, no pain for the patient, highly reproducible, and no loss of normal tissue	No pathological results, the vaginal stump lesions are not easily exposed
Zhang et al. [53]	VaIN I–III	60	Retrospective	93%		
Han et al. [54]	VaIN II–III	56	Prospective	88%		
Zhang et al. [55]	VaIN I–III	82	Prospective	90%		
**Surgical treatments**					Obtain pathology results that can help detect occult invasive vaginal cancer	Severe trauma, affecting quality of life
Zhang et al. [53]	VaIN I–III	40	Retrospective	82.5%		
**Intracavitary adiotherapy**					Satisfactory curative effect	Many adverse reactions, such as vaginal stenosis, vaginal ulcers and urinary tract symptoms
Song et al. [64]	VaIN I–III	34	Retrospective	88.2%		

### Medical treatements

The medical treatements for VaIN after hysterectomy contain imiquimod, 5-fluorouracil ointment, trichloroacetic acid, etc., which are suitable for patients who with persistent, multifocal disease, unable to undergo surgical treatment and without adverse medicine reactions [2].

Imiquimod, an immunomodulator, can produce interferon-α, interleukin-6, interleukin-8, interferon-γ, and other substances, thereby achieving antiviral and tumor treatment [40]. Studies have demonstrated that imiquimod has a total effective control rate of 57%–86% for VaIN. The local skin mucosal reaction is generally mild, with vaginal burning sensation and pain being the main adverse reactions [41, 42]. Inayama et al. conducted a meta-analysis and found that imiquimod can effectively treat VaINII–III, suggesting its potential in conservative treatment after hysterectomy [40]. Although local and systemic complications of imiquimod are common, discontinuation of treatment is rare [43].

5-Fluorouracil is a chemical exfoliation medicine used for treating local lesions by exfoliating the epithelium. However, it is not effective for deep or multi-focal lesions [44]. Studies have indicated that the success rate of 5-fluorouracil ointment in treating VaIN after hysterectomy ranges from 46 to 74% [45]. In addition, 5-fluorouracil has shown efficacy in the treatment of recurrent or persistent vaginal HSIL [46]. In addition, some patients may experience symptoms such as vaginal abnormal bleeding, pain, burning, and ulcers, which can result in poor compliance. However, these symptoms can be minimized by temporarily suspending treatment for 1 week and then resuming it at a dose of 2 g once a week for 10 to 12 weeks [47].

Trichloroacetic acid (TCA) is produced by chlorinating acetic acid. Research has indicated that TCA can cause varying degrees of damage to HPV DNA at different concentrations. A study involving 28 patients found that a 50% concentration of TCA could potentially be used as a treatment for vaginal LSIL after hysterectomy. This treatment option is cost-effective and easy to administer. The common side effects include pain and burning [48, 49].

### Physical therapy

CO_2_ laser treatment offers the benefits of easy operation and minimal complications. However, its effectiveness may be limited when it comes to treating concealed areas like the vaginal stump. Studies reported that laser therapy is suitable for addressing multifocal lesions in sexually active young women, with a remarkable cure rate of 90% and a recurrence rate of about 6.3% [38]. He et al. analyzed 116 VaIN patients who underwent laser therapy and were followed up for an average of 49.5 months and found 75% of patients experienced disease regression, while 23% experienced disease recurrence. After two laser treatments, the regression rate was 52.9%, and after three or more laser treatments, it was 26.5% [50]. Therefore, CO_2_ laser therapy is a safe and effective method for treating vaginal HSIL after hysterectomy. However, it is important to note the recurrence rate is high. Patients should be informed the risks of treatment failure, recurrence, and the necessary of long-term follow-up. Bogani et al. found there is no significant difference in the recurrence rate of VaIN between laser therapy and surgical resection. In addition, the risk of vaginal HSIL developing into invasive vaginal cancer after initial laser treatment was low [51]. Patients who are older than 50 years and underwent hysterectomy for CIN may be at a higher risk for VaINI + . Laser therapy is the independent prognostic factor that may prevent a second recurrence of VaINI +  [6].

Photodynamic therapy (PDT), a novel and targeted method for intraepithelial neoplasia, can concentrate photosensitizer at the site of the lesion and produce photochemical reaction when is irradiated with a specific wavelength, which can effectively eliminates diseased cells, damage tumor microvessels and activate the body’s immune system. PDT preserves the anatomical structure and physiological functions of the affected area. In the case of persistent HR-HPV infection after hysterectomy, 5-Aminolevulinic acid PDT represents a safe, non-invasive, and effective option [52]. Studies have reported that the HR-HPV remission rate of PDT for VaIN after hysterectomy ranges from 41.9% to 93.3% [53–55]. Wang et al. conducted a study on 163 patients with VAINI and HR-HPV infection, dividing them into two groups: the PDT group and the CO_2_ laser group. The PDT group received six ALA-PDT treatments, while the CO_2_ laser group received one CO_2_ laser treatment. The results indicated that the HPV clearance rate in the PDT group was 65.06%, which was significantly higher than that of the CO_2_ laser group. In addition, the VaINI-regression rate in the PDT group reached 95.18%, which was also significantly higher than that of the CO_2_ laser group [56]. PDT has shown to be highly effective in treating VaINI combined with HR-HPV infection [57]. Studies have indicated that the combination of CO_2_ laser and 5-ALA-PDT treatment resulted in a better HPV clearance rate without serious adverse events, making it a safe and effective method for treating VaIN [58].

Ultrasonic aspiration involves selectively tearing and aspirating high water content tissue, while preserving important structures. In addition, this technique provides tissue specimens for histological analysis [59]. Research has indicated that the recurrence rate of VaIN after hysterectomy in patients treated with ultrasonic aspiration ranges from 19.6% to 25% [60, 61]. This method is particularly suitable for aspirating tissue in challenging anatomical areas, such as the upper part of the vagina. It enables precise removal of diseased tissue with minimal damage to surrounding tissues.

### Surgical treatments

Surgical treatment options for VaIN after hysterectomy include local lesion resection, partial or total vaginectomy, and circular electroresection. The choice of treatments depends on the location and extent of the lesion. Laparoscopy, laparotomy, or transvaginal partial vaginal resection can be selected for lesions in the upper 1/3 of the vagina, while transvaginal partial vaginectomy is suitable for lesions in the lower 1/3. Surgical treatments yield pathological results that aid in the identification of concealed invasive vaginal cancer. However, these treatments also pose risks including bleeding, bladder or rectum damage, vaginal shortening, and stenosis, which may adversely affect the patients’ quality of life [62]. The success rate of surgical resection for VaIN after hysterectomy ranges from 66 to 83% [33, 39]. Zhang et al. reported an overall complete response rate of 82.5% for VaIN patients who underwent local surgical resection. At 12 months and 2 years of follow-up, the HPV clearance rates were 60% and 64.52%, respectively [53]. For potentially high-risk cases that invasive disease cannot be ruled out such as VaIN III, previous hysterectomies for HPV-related disease, menopausal women and so on, surgical excision should be the first treatment option [2, 31].

### Intracavitary radiotherapy

It is widely believed that the vagina is resistant to radiation and the upper part of the vagina can tolerant a higher radiation dose than the lower part. Patients with VaINIII after hysterectomy appear to benefit from radiotherapy, although it can lead to long-term distress due to complications such as fibrosis, vaginal stenosis, and sexual dysfunction. Therefore, determining appropriate dose limits for the vagina can greatly improve the quality of life for patients [63]. High-dose intracavitary brachytherapy is an effective treatment method for VaIN after hysterectomy, yielding a high cure rate. However, it may also cause vaginal toxicity and pose challenges in radiation technology [64]. The success rate of brachytherapy in treating VaIN after hysterectomy ranges from 71.4% to 90%, with a maximum sustained rate of 5.8% and a recurrence rate of 20% [38]. Some studies have suggested that pelvic radiation therapy is associated with an increased risk of secondary malignant tumors, so patients should be fully informed about their treatment options [65].

## Recurrence and follow-up

Similar to CIN, some VaIN can regress naturally. The lighter the disease, the greater the chance of regression. While most patients with VaIN II and III can be cured after treatment, a small number may experience relapse or progression to invasive cancer, necessitating long-term follow-up. A study by Monti et al. found that the recurrence rate of vaginal HSIL after treatment was 17%, with a 5-year cumulative recurrence rate of 30.4% and a median recurrence time of 15.5 months [66]. VaIN after hysterectomy still has a high risk of recurrence and progression after initial treatment. High-risk groups for invasive cancer progression include patients with VaIN III, those previously treated for CIN, and cervical cancer. Factors that increase the risk of recurrence include multifocal lesions, persistent HPV infection after following treatment of the primary disease, immunosuppressive status, smoking, etc. among others. Therefore, it is crucial to ensure that patients adhere to close follow-up [67]. The initial evaluation should include cytology at 6 months and HPV testing within 2 years, followed by annual screenings that include cytology, HPV, and colposcopy [2]. Patients with a history of CIN are more prone to developing VaIN after hysterectomy compared to those without a history of CIN. It is recommended that patients with a history of CIN undergo TCT and HPV screening every year for life after hysterectomy [17].

## Summary

VaIN after hysterectomy is a rare intraepithelial neoplasia of the female lower reproductive tract. The occurrence of VaIN is strongly associated with HR-HPV infection. Most patients with VaIN after hysterectomy do not exhibit specific clinical symptoms. The lesions are predominantly found in the upper vagina and have a multifocal distribution. Diagnosis of VaIN can be clearly established through cytology, HPV testing, colposcopy, and biopsy. It is recommended to routinely perform comprehensive biopsy under colposcopy to determine the extent and severity of the lesions and reduce the occurrence of vaginal stump lesions. Currently, the diagnosis and treatment of VaIN after hysterectomy should be individualized. It is important to address the potential impact of treatment on quality of life, as it may lead to psychological and psychosexual problems. Treatment for VaIN II and III in the stump vagina has a high response rate, but the recurrence rate is also high. This may be attributed to factors such as the unique anatomical structure of the stump vagina after hysterectomy, the multifocal nature of the disease, and limited understanding of the natural course and outcome. Long-term follow-up observation is necessary to determine if and when VaIN progresses to vaginal invasive cancer. Vaccination against HPV is recommended to prevent the development of HPV-related precancerous lesions.
